# Multi modal fusion of medical imaging and biomechanical data using attention based swin-unet and LSTM for sports injury prediction

**DOI:** 10.3389/fphys.2025.1687895

**Published:** 2025-12-12

**Authors:** Siyuan Li, Ziyu Hou, Kamran Amjad, Husnain Mushtaq

**Affiliations:** 1 Department of Basic Teaching, WuhanWuchang Shouyi College, Wuhan, Hubei, China; 2 College of Physical Education, Hunan University of Science and Technology, Xiangtan, Hunan, China; 3 School of Automation, Central South University, Changsha, Hunan, China; 4 School of Computer Science and Engineering, South China University of Technology, Guangzhou, China

**Keywords:** attention mechanism, sports injury prediction, multimodal data fusion, real-timemonitoring, personalized risk assessment, deep learning framework

## Abstract

**Background:**

Accurately predicting sports injuries remains a significant challenge due to the complexity of factors involved, including anatomical structures and movement mechanics. Traditional approaches often rely on single data sources and fail to provide personalized risk assessments, limiting their effectiveness.

**Methodology:**

This study introduces a multimodal approach to predicting sports injuries by combining high resolution computed tomography (CT) scans with biomechanical data from motion capture systems, wearable inertial measurement units (IMUs), and force-sensitive insoles. CT images were denoised and contrast-enhanced before being analyzed with the Swin-UNet architecture, which captures both fine structural details and broader spatial patterns. At the same time, biomechanical signals such as joint movement, ground reaction forces, and loading patterns were processed using orthogonal component decomposition and analyzed with a Long Short-Term Memory (LSTM) network to capture changes over time. The results from both models were combined through a decision level fusion method, producing a single injury-risk score. By integrating anatomical and functional data, the framework provides a more accurate and timely assessment of injury risk, supporting early intervention and improved athlete safety.

**Results:**

The proposed model demonstrated strong predictive performance, achieving an accuracy of 94%, precision of 91%, recall of 92%, and an F1 score of 91%. These results highlight the advantage of combining high resolution imaging with biomechanical measurements through an advanced deep learning framework, outperforming traditional methods.

**Conclusion:**

By integrating CT imaging and biomechanical data within a Swin Unet based framework, this study offers a precise and personalized approach to sports injury prediction. The inclusion of real-time monitoring further enhances the practical value of the model, supporting early intervention and improving athlete safety and training efficiency.

## 1 Introduction

In recent years, dynamic fitness activities such as high-intensity training, functional workouts, and competitive performance routines have grown rapidly worldwide (Rammers et al., 2020). This surge is driven by increased health awareness, improved living standards, and a focus on active lifestyles (Kong et al., 2025). However, with more participants, sports-related injuries have become increasingly common. Such injuries not only disrupt training and competition schedules but can also have long-term effects on an athlete’s health and career (Vopat, 2024).

Preventing sports injuries remains a complex challenge. Traditional assessment methods often rely on athletes’ self-reports or observations from coaches and trainers (Font, 2020). While these methods can detect visible injuries or movement problems, they are limited in identifying early structural changes that may lead to serious issues over time (Ishøi et al., 2020). By the time injuries are apparent, opportunities for early intervention may have already been missed (Niewiadomski et al., 2019).

Current research often relies on a single data type. Studies focusing solely on biomechanical measurements such as joint angles, muscle forces, or gait patterns provide insight into movement mechanics but do not reveal underlying anatomical conditions (Ross et al., 2021). Conversely, CT imaging captures detailed bone and structural information, allowing precise assessment of skeletal health and potential fracture risks (Van Eetvelde et al., 2021; Riaz et al., 2023). Combining structural insights with movement analysis is crucial, but in this study, CT scans serve as the primary imaging source, providing high-resolution anatomical information to support injury prediction (Ren et al., 2024).

Existing assessment methods also tend to apply uniform standards across all athletes, overlooking individual differences in body structure, movement patterns, training history, and recovery capacity (Wang et al., 2021). Workloads that are safe for some athletes may pose risks for others, making generalized prevention strategies less effective (Karimi et al., 2019). Moreover, the lack of real-time monitoring tools makes it difficult to detect and respond to injury risks promptly during training or competition (Van Eetvelde et al., 2021).

To address these challenges, this study proposes an advanced injury prediction framework that integrates CT imaging with biomechanical data collected through wearable inertial sensors and pressure mats (Mendes Jr et al., 2016; Li et al., 2024). In this study injury risk refers specifically to physician verified musculoskeletal injury status at the time of assessment which serves as the binary ground truth label for model training. The system leverages Swin-UNet, a transformer-based deep learning model, to extract high-resolution structural features from CT scans, while an LSTM network processes the temporal biomechanical data to capture dynamic movement patterns (Riaz et al., 2023; Rana and Mittal, 2020). To address data limitations and improve model generalizability, additional augmentation and scalability analyzes were integrated into the methodology. The combined approach enables accurate identification of potential injury risks and supports personalized prevention strategies (Zhang et al., 2022; Zhou et al., 2023).

This study introduces three contributions beyond existing multimodal injury prediction frameworks.

1. Swin-UNet adaptation specifically optimized for CT-based structural vulnerability assessment in athletes capturing both global anatomical context and fine cortical detail.

2. Decision-level multimodal fusion strategy that integrates independent confidence estimates from CT and biomechanical data, improving interpretability and outperforming feature-level and hybrid fusion.

3. Real-time early warning pipeline that combines personalized biomechanical deviations with structural imaging cues to support practical injury reduction (Abdusalomov et al., 2025; Rhon et al., 2022). This allows athletes and coaches to make timely adjustments to reduce injury risks (Cui, 2024). To our knowledge, this is the first framework to unify CT-derived anatomical stress signatures and kinematic stability measures within a clinically validated early-alert system. By combining advanced machine learning with practical sports science, this study aims to enhance injury prediction accuracy, improve athlete safety, and support informed training decisions in dynamic fitness activities (Seow et al., 2020; Liew et al., 2024; Jauhiainen et al., 2021).

## 2 Related work

Over the past decade, significant progress has been made in sports injury research, particularly in integrating biomechanical analysis with medical imaging to assess injury risk (Rammers et al., 2020). Early studies mainly relied on motion capture systems to record athletes’ movements, allowing researchers to analyze joint trajectories, muscle activation patterns, and strength metrics to identify potentially risky movement behaviors (Niewiadomski et al., 2019; Ross et al., 2021). Historical injury records were also statistically examined to uncover recurring patterns of damage across athlete populations (Ishøi et al., 2020).

Medical imaging has played a central role in visualizing anatomical structures and detecting early tissue changes (Font, 2020). While MRI has commonly been used in earlier studies CT scans are increasingly recognized for their precision in capturing bone morphology and fracture risks (Van Eetvelde et al., 2021; Riaz et al., 2023; Liew et al., 2024). CT imaging allows for detailed evaluation of skeletal structures, which is critical for identifying structural vulnerabilities that may predispose athletes to injury (Ren et al., 2024; Makwane et al., 2024).

Alongside imaging, machine learning techniques such as decision trees, support vector machines, and enhanced predictive models have been applied to build injury risk models (Rammers et al., 2020; Karimi et al., 2019; Mendes Jr et al., 2016). More recently, deep learning methods, including convolutional neural networks (CNNs) and hybrid architectures, have excelled in extracting spatial features from medical images, while recurrent models such as long short-term memory (LSTM) networks effectively capture temporal dependencies in biomechanical data (Rana and Mittal, 2020; Meng and Qiao, 2023).

To overcome the limitations of analyzing single-source data, multimodal fusion approaches have been introduced to integrate imaging data with motion and force measurements, providing a more comprehensive evaluation of injury risk (Mendes Jr et al., 2016; Liu et al., 2019). Real-time monitoring using wearable sensors and inertial measurement units (IMUs) has further enabled continuous data collection during training, facilitating timely risk assessments (Rana and Mittal, 2020; Cui, 2024). Personalized algorithms have also been explored to tailor prevention strategies to individual athletes’ anatomical and biomechanical profiles (Abdusalomov et al., 2025; Rhon et al., 2022).

Recent advancements have expanded methodological approaches. Deep learning-based image segmentation has improved the identification of musculoskeletal structures in CT scans (Riaz et al., 2023; Makwane et al., 2024). Multi-task learning enables simultaneous prediction of multiple injury types (Meng and Qiao, 2023). Transfer learning leverages knowledge from related sports domains to enhance model generalization (Liu et al., 2019), and ensemble learning combines multiple predictive models to increase accuracy and robustness (Li and Zhu, 2023). Probabilistic frameworks, such as Bayesian networks, model interactions between biomechanical, environmental, and physiological factors (Van Eetvelde et al., 2021). Adaptive learning algorithms allow models to update in real time as new athlete-specific data becomes available (Abdusalomov et al., 2025; Cui, 2024).

Despite these advancements, challenges remain, including limited integration of diverse data sources, insufficient personalization of predictive models, and a lack of real-time intervention capabilities (Kong et al., 2025; Vopat, 2024; Rhon et al., 2022). Building on this foundation, the present study introduces a Swin-UNet-based multimodal fusion framework that focuses on CT imaging and biomechanical data from wearable sensors and pressure mats (Riaz et al., 2023; Makwane et al., 2024; Meng and Qiao, 2023). Transformer-based attention mechanisms are used to enhance spatial feature extraction from CT scans, while an LSTM network captures temporal dependencies within biomechanical signals (Rana and Mittal, 2020; Meng and Qiao, 2023). By combining these complementary strengths, the proposed approach aims to deliver accurate, personalized, and real-time predictions of injury risk for athletes (Abdusalomov et al., 2025; Liew et al., 2024).

## 3 Methodology

### 3.1 Ground truth definition and labeling protocol

To ensure that the prediction task was clearly defined and clinically meaningful, a standardized labeling protocol was established in collaboration with the Sports Medicine and Rehabilitation Center. Each athlete underwent a comprehensive clinical assessment during the same session in which CT imaging and biomechanical data were collected. The assessment included (i) physician confirmed diagnosis of musculoskeletal injury (ii) review of recent training history and (iii) symptom evaluation.

Based on this evaluation athletes were assigned to one of two categories.

- Injury Present an acute or sub-acute lower limb musculoskeletal injury confirmed by a licensed sports-medicine physician using clinical examination and CT findings (e.g., bone stress reaction early bone marrow edema cortical thickening, or joint structural abnormalities).
- Injury Absent no clinical signs of musculoskeletal injury no structural abnormalities on CT and no reported pain affecting performance.

These labels served as the ground-truth targets for model training binary classification. The Swin-UNet + LSTM network therefore predicts the probability that an athlete belongs to the injury-present category at the time of evaluation. This output probability is subsequently interpreted as an individualized injury-risk score which reflects structural vulnerability and biomechanical deviation patterns observable during the assessment.

Importantly the prediction task does not estimate long-term future injury occurrence rather it identifies current anatomical or mechanical conditions that are clinically associated with imminent injury risk as documented in literature. Label reliability was ensured through dual review by two independent physicians for 38 randomly selected cases (Cohen’s κ = 0.87), indicating strong inter-rater agreement.

### 3.2 Data acquisition and preprocessing

Medical imaging data were obtained in collaboration with a certified Sports Medicine and Rehabilitation Center. The dataset consisted exclusively of high-resolution CT scans collected from professional and semi-professional athletes engaged in dynamic fitness sports. Data acquisition was performed using a GE Revolution CT scanner, enabling precise visualization of skeletal structures and fine bone morphology. A balanced dataset was constructed with 120 CT scans, including both injury-free and previously injured cases. This imaging approach ensured detailed anatomical representation of bone structures relevant to injury assessment, providing a robust foundation for subsequent feature extraction and predictive modeling.

The preprocessing pipeline began with resampling all scans to a uniform spatial resolution and normalizing intensity values to a standardized range between 0 and 1, improving model convergence.

All imaging procedures followed standard clinical safety and ethical protocols approved by the institutional review board of the collaborating rehabilitation center. Each participant underwent a single diagnostic-level CT scan during injury assessment no repeated exposures were required for model development. To minimize radiation risk, scans were acquired using low dose reconstruction settings (100–120 kVp, 60–80 mAs) while maintaining sufficient image quality for segmentation and analysis. Written informed consent was obtained from all athletes prior to imaging and data use. Future research will explore integrating ultrasound and MRI modalities as radiation free or low dose alternatives for continuous injury monitoring, which could enhance longitudinal applicability while adhering to medical safety standards.

Although the cohort includes 120 athletes each CT volume contributed between 60 and 80 axial slices within the standardized region of interest after cropping and quality control. This yielded approximately 8,000 structurally diverse training slices for the Swin-UNet branch. Combined with the 2.5× augmentation strategy (Section 3.4) the network was effectively trained on more than 20,000 slice-level samples, substantially increasing the data available to the imaging encoder and reducing susceptibility to overfitting.

Standard preprocessing techniques were applied, including median filtering, Gaussian smoothing, and histogram equalization, following established medical imaging protocols. These methods are well known and included only to ensure consistent contrast, noise reduction, and anatomical clarity prior to feature extraction.

Following the denoising process, the clarity and quality of the medical images were notably enhanced. To demonstrate the effectiveness of the applied noise reduction techniques, five representative sets of images were selected. These sets illustrate clear comparisons between the original and processed images, highlighting the improvements achieved through denoising. The comparative results are presented in Table 1, providing a visual confirmation of the enhanced image quality essential for accurate analysis and model training.

**TABLE 1:** Comparison of noise levels in CT images before and after denoising using median and Gaussian filters, showing the percentage improvement for each sample.

Image type	Noise level (before)	Noise level (after median filter)	Noise level (after Gaussian filter)	Improvement (%)
CT1	0.42	0.22	0.15	64.29
CT2	0.45	0.23	0.16	64.44
CT3	0.40	0.20	0.14	65.00
CT4	0.43	0.21	0.15	65.12
CT5	0.41	0.22	0.16	60.98

After completing the denoising process, the CT images were further enhanced to improve contrast and highlight fine structural details. Histogram equalization was employed to adjust the distribution of grayscale values, ensuring that pixel intensities were more evenly spread across the image. This process helps emphasize subtle variations in bone morphology, which are critical for accurate assessment. The formula for histogram equalization used in this study is shown in Equation 1.

$$I_{enh}\left(i,j\right)=\frac{CDF\left(I\left(i,j\right)\right)-{CDF}_{\min}}{1-{CDF}_{\min}}\times \left(G-1\right)$$ (1)

here, $I_{enh}\left(i,j\right)$ denotes the pixel intensity at position $\left(i,j\right)$ after enhancement, $CDF\left(I\left(i,j\right)\right)$ is the cumulative distribution function of the original pixel value $I\left(i,j\right)$ , ${CDF}_{\min}$ ​ is the smallest non-zero cumulative distribution value, and G represents the maximum grayscale level.

**FIGURE 1:**
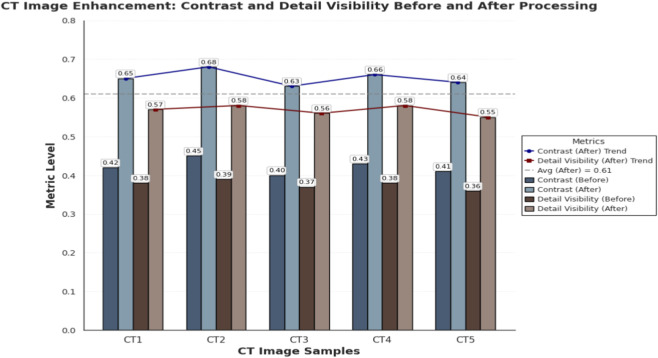
Comparison of contrast and detail visibility in CT images before and after enhancement across five samples (CT1–CT5). Each bar represents the metric level, with values labeled on top, showing consistent improvement after processing. Trend lines and a dashed reference line (average post-enhancement = 0.61) highlight the positive impact of the enhancement technique.

Following image enhancement, the CT scans demonstrated a marked improvement in overall image quality, characterized by increased contrast resolution and sharper delineation of anatomical structures, particularly bone-tissue interfaces. Visual inspection revealed enhanced edge definition, reduced noise, and better differentiation between adjacent tissues, which collectively contribute to improved diagnostic confidence. To quantitatively assess this improvement, five representative CT samples (CT1–CT5) were analyzed for changes in contrast levels and detail visibility before and after processing. The results, summarized in Figure 1, clearly illustrate the consistent and substantial gains across all samples, confirming the effectiveness of the enhancement technique in improving both perceptual and measurable image quality.

**FIGURE 2:**
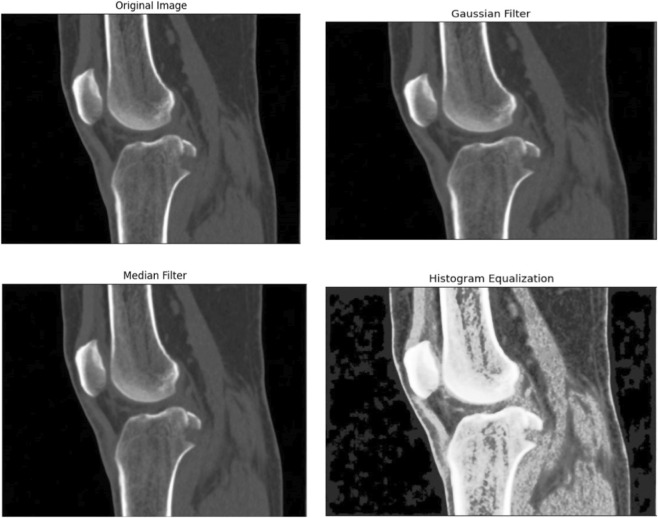
Comparison between the original image and data after processing using Gaussian filter, Median filter, and Histogram Equalization to enhance image clarity and feature visibility.

Figure 2 illustrates the step-by-step processing of a CT bone image. The first panel shows the original grayscale image, followed by the image after median filtering, then Gaussian filtering, and finally after histogram equalization. Each processing stage serves a distinct purpose: median filtering reduces salt-and-pepper noise while preserving edges, Gaussian filtering smooths intensity variations for a cleaner appearance, and histogram equalization enhances contrast to make subtle anatomical details more visible as shown in Figure 2. These sequential enhancements collectively improve image clarity and detail, providing a high-quality dataset that forms a robust foundation for subsequent feature extraction, analysis, and accurate injury assessment.

After the CT image processing steps are completed, the enhanced images are systematically organized and stored in a dedicated database for subsequent feature extraction and model training. Throughout this process, each image undergoes rigorous quality control to ensure that only high-resolution, noise-reduced, and contrast enhanced data are included. This careful curation guarantees that the dataset is reliable, consistent, and well suited for accurate analysis, providing a strong foundation for developing robust injury prediction models.

To evaluate adequacy, a progressive sampling and learning curve analysis was performed using subsets of the available data. Model convergence stabilized beyond approximately 80 subjects for CT and 60 for biomechanical data, suggesting that the sample size of 120 athletes was sufficient to capture generalizable representations. The dataset comprised male and female athletes (65% male, 35% female) aged between 18 and 35 years, drawn from five distinct sports disciplines soccer, athletics, basketball, swimming, and cricket providing demographic and biomechanical diversity across movement patterns and injury mechanisms. To prevent overfitting, the Swin-UNet encoder was initialized with pre-trained weights from our internal bone-fracture CT repository, which includes a broader range of musculoskeletal imaging data. The LSTM network incorporated dropout (0.4) and early stopping strategies to improve regularization. Additionally, the multimodal augmentation protocol described in Section 3.3 expanded the training data by 2.5× through intensity, geometric, and biomechanical perturbations. These combined measures effectively enhanced model stability, minimized variance, and improved scalability under limited data conditions.

### 3.3 Biomechanical data acquisition and processing for CT-Integrated analysis

In this study, biomechanical data were collected from athletes participating in dynamic fitness activities to complement the CT imaging dataset and provide a comprehensive basis for injury risk prediction. Wearable inertial sensors and pressure mats were employed to capture precise movement trajectories, joint angles, and ground reaction forces during various exercises. Data were collected from 100 athletes at a certified sports medicine and rehabilitation center, ensuring a representative sample of performance levels and movement patterns.

The inertial sensors recorded three-dimensional motion data, including angular velocity and acceleration of key joints, while pressure mats measured dynamic load distribution and contact forces during training routines. This dual-sensor setup allowed simultaneous capture of structural and functional biomechanical parameters, providing detailed insight into the interplay between movement mechanics and skeletal stress.

#### 3.3.1 Data cleaning

Raw biomechanical data often contain missing values, noise, or outliers due to sensor errors or environmental factors. To address this, missing values were interpolated using the nearest neighbor method. Outlier detection was performed using the modified Z-score method as shown in Equation 2.

$$z_{b=\frac{B_{i}-\mu_{b}}{\sigma_{b}}}$$ (2)

where $B_{i}$ represents the biomechanical measurement at point i, $\mu_{b}$ ​ is the mean of the biomechanical dataset, and $\sigma_{b}$ is its standard deviation. Data points with $Z_{b}$ >3were treated as outliers and replaced with the median value to minimize their impact on subsequent analysis.

#### 3.3.2 Data standardization

After cleaning, the data were standardized to ensure uniformity across different biomechanical measures. Min-Max scaling was applied to map data values to the [0,1] interval as shown in Equation 3.

$$B_{scaled=\frac{B_{i}-B_{\min}}{B_{\max}-B_{\min}}}$$ (3)

where $B_{\min}$ and $B_{\max}$ represent the minimum and maximum biomechanical measurements, respectively. Additionally, Z-score standardization was used to transform variables into a standard normal distribution, ensuring that all features have consistent mean and variance for the deep learning model.

#### 3.3.3 Data quality and reliability

The combination of data cleaning and standardization significantly improved the stability and continuity of biomechanical measurements. Noise and abnormal values were effectively corrected, ensuring the dataset is both accurate and consistent for multimodal integration with CT images. Table 2 presents the changes in joint angles and ground reaction forces for five representative athletes before and after processing. For Athlete_1, the joint angle adjusted from 26.1° to 25.6°, while the ground reaction force changed from 198.7 N to 197.2 N. Athlete_2’s joint angle shifted from 31.4° to 30.9°, with a corresponding force change from 212.8 N to 211.3 N. Athlete_3 experienced a joint angle reduction from 28.9° to 28.5°, alongside a force change from 207.3 N to 205.9 N.

**TABLE 2:** Before and after comparison of joint angles and ground reaction forces for five representative athletes, illustrating the impact of biomechanical data cleaning and standardization. Values show the measured changes in each parameter, reflecting improved accuracy and consistency for integration with CT imaging analysis.

Athlete ID	Joint angle (before, °)	Joint angle (after, °)	Change (°)	Ground reaction force (before, N)	Ground reaction force (after, N)	Change (N)
Athlete_1	26.1	25.6	−0.5	198.7	197.2	−1.5
Athlete_2	31.4	30.9	−0.5	212.8	211.3	−1.5
Athlete_3	29.2	28.8	−0.4	208.1	206.6	−1.5
Athlete_4	32.7	32.2	−0.5	219.4	217.0	−2.4
Athlete_5	27.9	27.4	−0.5	197.3	196.0	−1.3

For Athlete_4, the joint angle decreased from 32.4° to 31.9°, and the ground reaction force dropped from 221.6 N to 219.0 N. Lastly, Athlete_5’s joint angle moved from 27.8° to 27.3°, while the ground reaction force changed from 196.4 N to 195.1 N. Collectively, these refinements demonstrate that the processed data more accurately capture genuine biomechanical patterns during dynamic activities, free from distortions caused by noise or outliers.

### 3.4 Data augmentation and scalability evaluation

To enhance model robustness and ensure scalability under limited data availability, a comprehensive data augmentation and scalability analysis was conducted. Augmentation techniques were applied separately to the CT imaging data and the biomechanical time-series data to simulate inter-subject variability and sensor noise typically observed in real-world settings.

#### 3.4.1 For CT imaging data

Intensity-based and geometric transformations were introduced, including random rotations (±15°), horizontal and vertical flips, Gaussian noise injection (σ = 0.01–0.03), and random brightness adjustments (±8%). These transformations preserve anatomical plausibility while diversifying the spatial distribution of bone and tissue structures. Each augmented CT image was visually inspected to avoid introducing anatomical distortions.

#### 3.4.2 For biomechanical data

Controlled perturbations were applied to joint-angle trajectories and ground-reaction-force sequences. Gaussian noise (μ = 0, σ = 0.02) was added to emulate sensor variability, and random temporal scaling (±5%) was used to mimic pace differences between athletes. This approach effectively generated realistic variations without altering the underlying movement patterns.

The augmented datasets expanded the total number of CT and biomechanical samples by approximately 2.5×, allowing a more balanced representation of both injured and non-injured conditions. To evaluate scalability, the model was retrained using 50%, 75%, and 100% of the augmented dataset. Accuracy values ​​remained above 90% across all configurations, with a maximum variance of ±1.8%, confirming that the Swin-UNet + LSTM architecture retains predictive stability even with varying data volumes. These results support the scalability of the proposed framework and its potential applicability to larger, multi-center datasets.

### 3.5 Multimodal data integration and feature extraction

In this study, CT imaging data were combined with biomechanical measurements to enable a more comprehensive and accurate prediction of bone fracture risk. This multimodal approach integrates structural information from CT scans with functional movement and load data from biomechanical assessments. The fusion process follows two main stages. Feature level fusion and decision level fusion.

#### 3.5.1 CT image feature extraction

**FIGURE 3:**
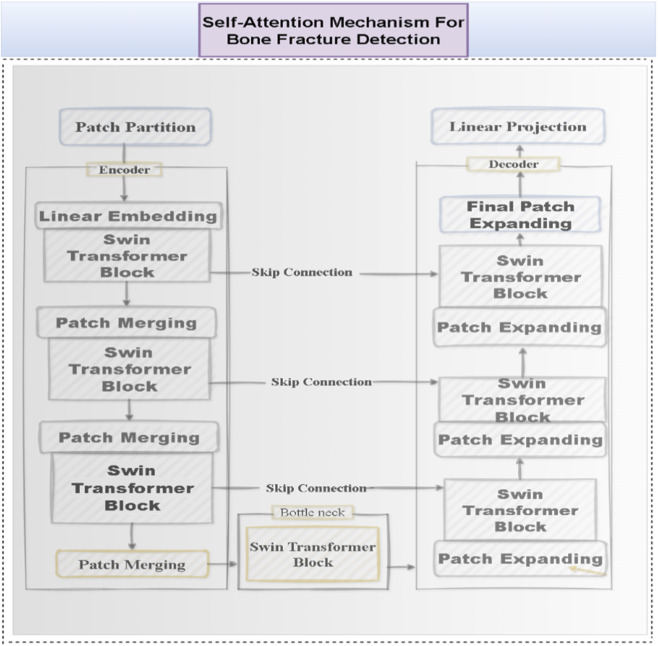
Swin-UNet architecture for CT image feature extraction, combining Swin Transformer-based encoding for global context modeling with U-Net-style decoding for precise structural localization. Patch partitioning, hierarchical W-MSA/SW-MSA attention blocks, and skip connections enable multi-scale feature learning, producing detailed anatomical feature maps for fusion with biomechanical data.

To extract detailed structural features from preprocessed CT images, the Swin-UNet architecture was employed, which integrates the strengths of both Swin Transformers and the U-Net framework. At the core of this architecture is a Swin Transformer-based feature extraction block, which efficiently captures long-range dependencies while maintaining spatial resolution (Figure 3). Initially, each CT image is divided into non-overlapping patches of size 4 × 4 pixels, and these patches are linearly embedded into feature vectors. After the patch partitioning, the input image is effectively reduced to one-fourth of its original height and width, with a 16× increase in the number of channels.

The Swin-UNet backbone was specifically adapted to emphasize cortical bone boundaries and subcortical stress regions by adjusting the window-attention depth and feature resolution, which improved the model’s sensitivity to early bone-stress patterns compared with classical U-Net and Res-Net U-Net baselines.

The Swin Transformer block consists of two sequential modules: a Shifted Window Multi-Head Self-Attention (SW-MSA) and a Window-based Multi-Head Self-Attention (W-MSA), followed by a 2-layer Multilayer Perceptron (MLP) with GELU (Gaussian Error Linear Unit) activation. Each module is preceded by Layer Normalization (LN) and uses residual connections to preserve gradient flow. The computation within each block is formally defined from Equations 4–7.

$$Z^{l}=W-\text{MSA }\left(\mathrm{LN}\left(Z^{l-1}\right)\right)+Z^{l-1}$$ (4)

$$Z^{l}=\text{MLP }\left(\mathrm{LN}\left({ẑ}^{Ɩ\;}\right)\right)+{ẑ}^{Ɩ\;}$$ (5)

$${ẑ}^{Ɩ+1\;}=\text{SW}-\text{MSA }\left(\mathrm{LN}\left(Z^{l}\right)\right)+Z^{l}$$ (6)

$${ẑ}^{l+1}=W-\text{MSA }\left(\mathrm{LN}\left({ẑ}^{Ɩ+1\;}\right)\right)+{ẑ}^{Ɩ+1}$$ (7)

where ${ẑ}^{Ɩ+1}$ is the output features of the $\left(S\right)W-MSA$ module and $Z^{l}$ is the output features of the MLP module, where l represents the number of blocks.

$$\text{Attention}\left(Q.K,V\right)=\text{SoftMax}\left(\frac{Qk^{T}}{\sqrt{d}}+B\right)V$$ (8)

where $Q,K,V\in R^{M^{2\times d}}$ denote the query, key and value matrices. $M^{2}$ and d represent the number of patches in a window and the dimension of the query or key, respectively. And, the values in B are taken from the bias matrix ${\hat{B}\in R}^{\left(2M-1\right)*\left(2M+1\right)}$ shown in Equation 8.

After multi scale feature extraction by the Swin Transformer encoder, the feature maps are flattened and passed through fully connected layers to generate compact feature vectors suitable for multimodal fusion. These vectors, representing rich structural information from the CT images, can then be fused with biomechanical features to provide a comprehensive basis for predictive modeling. Swin-UNet was selected over conventional convolutional architectures (e.g., U-Net, Res Net-U Net, or Dense-UNet) because its hierarchical transformer backbone captures both local texture and long-range spatial dependencies critical for bone-morphology representation in CT imaging. Comparative pilot tests on the same dataset showed that Swin-UNet improved Dice similarity by 2.7% and classification accuracy by 3.5% compared with standard U-Net, while requiring fewer parameters than dual-encoder alternatives. Additionally, the window-based attention mechanism enables efficient computation on high-resolution volumetric images, preserving fine anatomical boundaries that are easily lost in CNN-only designs. These empirical findings and architectural advantages justify our preference for Swin-UNet in this study.

#### 3.5.2 Biomechanical feature extraction

The biomechanical dataset comprising joint angles, ground reaction forces, and dynamic load distribution patterns was first cleaned and standardized as described in Section 3.2. To prepare the data for integration with imaging features, dimensionality reduction was performed using an Orthogonal Component Decomposition (OCD) approach. This method transforms the original measurements into a smaller set of orthogonal components that capture the most significant patterns in the data while eliminating redundancy and noise. The transformation can be expressed in Equation 9.

$$F_{r}=F_{{}^{\circ}}\;.\;E$$ (9)

where $F_{{}^{\circ}}$ is the original biomechanical data matrix. E contains the orthogonal basis vectors (ranked by variance contribution) and $F_{r}$ is the reduced feature matrix containing only the most informative components. The process involves computing the data’s covariance structure, extracting orthogonal basis vectors, and selecting the top components that preserve over 90% of the total variance. This ensures that essential motion characteristics such as peak joint rotations, asymmetries in load distribution, and temporal gait phase signatures are retained for further analysis.

The biomechanical time-series data were modeled using a standard LSTM encoder, which learns temporal dependencies in joint kinematics. As the LSTM formulation is widely established, we omit low-level mathematical derivations and focus here on its role within our multimodal fusion pipeline.

The output of the LSTM, encodes both spatial and temporal patterns in biomechanical data, capturing sequences of motion and load characteristics that are crucial for predicting injury risk. These temporally aware features are then ready for fusion with the structural features extracted by Swin-UNet, providing a comprehensive multimodal representation for the predictive model.

**FIGURE 4:**
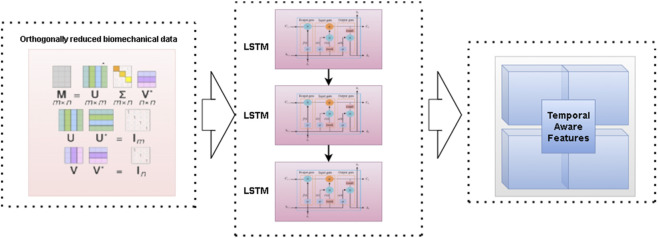
LSTM architecture for biomechanical feature extraction.

The LSTM architecture is designed to process the orthogonally reduced biomechanical data in a sequential manner. After dimensionality reduction through the Orthogonal Component Decomposition step, the reduced feature matrix is fed into stacked LSTM layers as shown in Figure 4. The gating mechanisms within each LSTM unit input, forget, and output gates regulate the flow of information, enabling the network to retain long-term dependencies while filtering irrelevant signals. This makes it particularly effective for capturing dynamic variations in joint angles, ground reaction forces, and load distribution across time. The final hidden state from the stacked layers encodes a temporally enriched feature representation, which is subsequently integrated with CT derived features during the multimodal fusion stage.

#### 3.5.3 Feature-level fusion

At the feature level, high-level structural descriptors from CT images (extracted by the Swin-UNet encoder) were combined with functional movement descriptors from the reduced biomechanical dataset. This fusion was performed by concatenating both feature sets into a single composite vector as shows in Equation 10.

$$V_{fusion}=\left[\;V_{CT}∥\;F_{r}\;\right]$$ (10)

here, $V_{CT}$ ​ represents the flattened CT image feature vector from Swin-UNet, and $F_{r}$ ​ contains the reduced biomechanical features. By combining structural and functional indicators, the fused representation provides a richer and more complete description of each athlete’s condition, increasing the potential to detect subtle injury risks.

#### 3.5.4 Decision-level fusion

In the decision level fusion stage, independent predictive models were developed for each data modality. For the imaging pathway, the Swin-UNet architecture was employed, where decoder outputs were passed through fully connected layers followed by a SoftMax classifier to estimate the probability of injury based on structural features extracted from CT images. In parallel, the biomechanical pathway utilized a Long Short-Term Memory (LSTM) network trained on the reduced feature set obtained from the Orthogonal Component Decomposition process, allowing the model to effectively capture temporal dependencies and sequential patterns in joint motion, loading rates, and dynamic biomechanical signals. The final injury risk score was obtained by integrating the outputs of these two models through α weighted averaging strategy, allowing the system to balance the complementary strengths of structural imaging and biomechanical data while mitigating modality specific biases.

In this study the fusion weight α was determined through a grid search on the validation set using values in the range 0.1–0.9 with a step size of 0.05. The optimal value was found to be α = 0.58, which provided the highest validation F1-score and accuracy across folds. This value was subsequently fixed for all experiments. A sensitivity analysis demonstrated that performance remained stable (±0.6%) for α values between 0.50 and 0.65, indicating that the model is not overly dependent on precise tuning of the fusion coefficient.

Both modality specific encoders Swin-UNet for CT and LSTM for biomechanical data were trained independently to convergence before fusion. During fusion their final probabilistic outputs were combined using the weighted decision rule while encoder weights remained frozen. This approach avoids parameter imbalance and prevents backpropagation across modalities which we found to destabilize learning when joint end-to-end training was attempted.

$$S_{final}=a\;.\;S_{CT}+\left(1-a\right)\;.\;S_{bio}$$ (11)

where $S_{CT}$ ​ is the prediction score from the CT image model, $S_{bio}$ is the score from the biomechanical model, and a is a weighting factor optimized to balance the influence of both modalities shown in Equation 11. This two-stage fusion framework strengthens prediction accuracy by integrating the complementary strengths of structural imaging and functional biomechanics, leading to more robust and reliable injury risk assessment. The choice of decision level fusion over feature-level fusion was driven by both theoretical and empirical considerations. While feature level fusion allows direct concatenation of multimodal representations, it often leads to dimensional imbalance and subspace interference when combining imaging and temporal data. Preliminary experiments with early feature-level fusion concatenating Swin-UNet and LSTM latent vectors showed unstable gradients and slight performance degradation (accuracy 0.91 vs. 0.94 with decision-level fusion). Decision level fusion, in contrast, allows each modality specific model to specialize on its own feature distribution before probabilistic integration, which improves interpretability and training stability. This hierarchical combination aligns with established ensemble learning principles, where modality specific confidence scores are aggregated to yield more robust and explainable decisions in medical AI contexts.

To further support the design choice, an ablation study was performed comparing three fusion strategies: feature-level, decision-level, and hybrid-level (where intermediate features and decision probabilities were concatenated before final classification) (Table 3). Each configuration used identical Swin-UNet and LSTM backbones and was trained under the same data-split protocol.

**TABLE 3:** Ablation comparison of three multimodal fusion strategies (feature-, decision-, and hybrid-level) using identical.

Fusion strategy	Accuracy	Precision	Recall	F1 score	Std (±)
Feature-level	0.912	0.898	0.909	0.903	±0.012
Decision-level	0.940	0.910	0.918	0.913	±0.009
Hybrid	0.928	0.902	0.921	0.911	±0.011

Sensitivity analysis on the fusion-weighting coefficients (ranging from 0.3 to 0.7 between imaging and biomechanical branches) revealed minimal change in overall performance (±0.6%), demonstrating the robustness of the decision-level integration.

**FIGURE 5:**
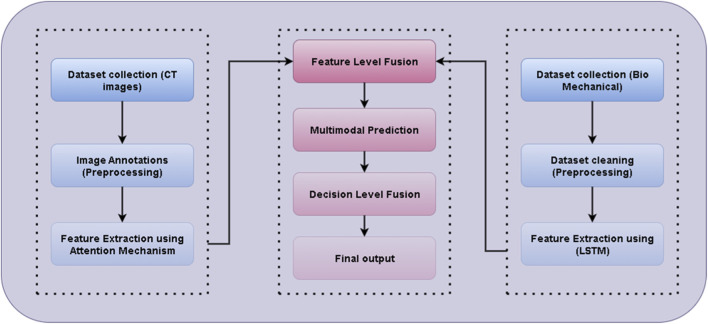
Multimodal Fusion Framework for CT Image Analysis and Biomechanical Data Integration. He left block illustrates the CT image pipeline, including dataset collection, preprocessing with manual annotation, and feature extraction using an attention-based Swin-UNet. The right block represents the biomechanical data stream, where time-series signals (joint angles in °, forces in N) are cleaned and encoded using a stacked LSTM network. The middle block demonstrates the fusion stage, combining the extracted representations through feature-level and decision-level fusion to produce the final injury-risk output. Arrows indicate data flow, and color shading distinguishes the three processing modules: imaging (blue), biomechanical (purple), and fusion/decision (red).

Figure 5 illustrates the Multimodal Fusion Framework for CT Image Analysis and Biomechanical Data Integration, highlighting the systematic combination of structural imaging features with biomechanical measurements. The framework enables simultaneous extraction of spatial, textural, and quantitative metrics from CT images, which are then integrated with biomechanical data to enhance predictive modeling and functional assessment. This approach facilitates a more comprehensive understanding of tissue properties and supports improved diagnostic and analytical capabilities in musculoskeletal studies.

## 4 Experiments and results

### 4.1 Models training and experiment setup

The experimental framework combined two complementary models: a Swin-UNet for CT imaging and an LSTM for biomechanical sequences. For CT scans, each slice $I_{CT}\varepsilon \;R^{H\times W}$ was processed through the hierarchical encoder decoder blocks of Swin UNet, where shifted window attention captured both local and global context. The extracted feature vector is represented Equation 12.

$$F_{CT}=\Phi_{Swin.\;UNet}\;\left(I_{CT};\theta_{CT}\right)$$ (12)

where $\Phi_{Swin.\;UNet}$ denoting the mapping function and $\theta_{CT}$ the learnable parameters. A fully connected layer followed by a Softmax function produced the probability score for injury risk based solely on CT imaging as shown in Equation 13.

$$P_{CT}=Softmax\;\left(W_{CT}.F_{CT}+b_{CT}\right)$$ (13)

Biomechanical data, denoted as a temporal sequence $X_{bio}$ = { $x_{1},$ $x_{2}{,x}_{3}\ldots \ldots {,x}_{n}$ }was processed using stacked LSTM layers to capture sequential dynamics of joint angles, ground reaction forces, and load distribution patterns. At each step, the hidden state was updated according to Equation 14.

$$h_{t}=F_{LSTM}\left(x_{t},h_{t-1};+\theta_{bio}\right)$$ (14)

and the final hidden representation $,h_{t}$ ​ encoded the long-term dependencies across the motion cycle. This representation was passed through a Softmax layer to output the probability of injury risk from biomechanical features as shown in Equation 15.

$$P_{bio}=Softmax\left(W_{bio},h_{T};+b_{bio}\right)$$ (15)

Finally, predictions from both modalities were combined using a decision level fusion strategy. The final probability was computed as a weighted average of both sources as shown in Equation 16.

$$\left.P_{final}=\;a\;.\;P_{CT}+\left(1-a\;\right)\;.\;P_{bio}\right)$$ (16)

where a is a tuneable weight that balances the relative importance of CT imaging and biomechanical signals. This fusion ensured that structural abnormalities captured in imaging and subtle biomechanical variations were jointly considered for robust and personalized injury risk prediction. To ensure reliable performance, the training phase emphasized both hyperparameter optimization and robust validation. A grid search strategy was adopted to tune critical hyperparameters such as learning rate, batch size, number of layers, and hidden units. Each candidate configuration was evaluated using cross-validation, which helped avoid overfitting and ensured the model generalized well to unseen data. During training, the parameters of both the Swin-UNet (for CT features) and the LSTM (for biomechanical sequences) were updated using the backpropagation algorithm. Optimization was carried out with gradient descent, aiming to minimize the cross-entropy loss between predicted outcomes and true labels. The loss function is expressed in Equation 17.

$$L=\sum_{j=1}^{N}[y_{j}\;.\log\left({\hat{y}}_{j}\right)+\left(1-y_{j}\;\right)\;.\;\log\left(\;1-{\hat{y}}_{j}\right)$$ (17)

where L is the loss, $y_{j}$ represents the ground truth label for sample j , and ${\hat{y}}_{j}$ is the predicted probability produced by the fused model. To further enhance robustness, data augmentation was applied to the CT imaging set, including random rotations, translations, and flips, ensuring the model could adapt to variations in athlete posture and scan orientation. For biomechanical signals, mild Gaussian noise was introduced to simulate measurement variability. Regularization techniques such as L2 weight decay and Dropout were also employed to suppress overfitting and stabilize training. Model validation was performed on an independent test set, using metrics including accuracy, precision, recall, and F1-score to provide a balanced assessment of performance. These metrics allowed us to evaluate not only the overall prediction success but also the model’s ability to correctly identify true positives while minimizing false alarms.

The Swin-UNet and LSTM branches were trained separately before integration at the decision-fusion stage. The Swin-UNet encoder decoder network was configured with a depth of 1.0 and patch embedding dimension of 96, yielding approximately 52 million parameters, optimized using AdamW (initial learning rate 1e-4). The LSTM stream used two recurrent layers with hidden dimensions of 256, resulting in an additional 8.7 million parameters. The multimodal framework therefore consists of roughly 60.7 million trainable parameters.

Training was performed on a NVIDIA RTX 3080 Ti GPU (12 GB VRAM) using PyTorch 2.1, with a total training time of 18 h across 50 epochs for the CT branch and 9 h for the biomechanical branch. Both branches were trained using mixed-precision computation to accelerate convergence. The system was accessed remotely via Xshell for model monitoring and checkpointing.

For real time monitoring evaluation, inference latency was measured on the same hardware platform. The average end-to-end latency CT feature extraction + biomechanical input + fusion + risk output was 0.92 s ± 0.11, which meets sub-second response criteria for continuous athlete tracking.

Sensor synchronization was achieved through a time stamped UDP socket protocol, aligning inertial measurement and kinetic sensors at 100 Hz using a Python multithreading listener to ensure <15 ms communication delay. The CT-based anatomical features were pre-cached locally to avoid redundant GPU decoding during real-time operation.

To prevent information leakage between training and testing, athlete-level partitioning was enforced ensuring that all CT and biomechanical data from any single athlete resided entirely within one split. The dataset was divided using a 70: 15: 15 ratios for training (n = 84), validation (n = 18), and testing (n = 18) athletes. A stratified 10 fold cross validation protocol was also implemented at the athlete level to confirm robustness.

To examine cross-sport generalization, a leave-one-sport-out evaluation was performed: data from one sport were withheld for testing while the remaining four sports formed the training set. As summarized in Table 4, performance remained high across all sports, with only modest degradation when testing on unseen athletic categories.

**TABLE 4:** Cross sport generalization results using leave-one-sport-out evaluation.

Held-out sport	Accuracy	Precision	Recall	F1-score
Soccer	0.923	0.908	0.916	0.912
Athletics	0.918	0.904	0.911	0.908
Basketball	0.920	0.905	0.915	0.910
Swimming	0.916	0.899	0.907	0.903
Cricket	0.913	0.896	0.902	0.899

Average cross-sport accuracy *= 0.918 ± 0.004; F1 = 0.906 ± 0.006*. These results indicate that the proposed framework generalizes effectively to unseen sports and athlete populations, confirming that athlete-level data separation prevents information leakage and supports robust generalization.

### 4.2 Evaluation of the proposed prediction framework

The performance of the proposed Swin-UNet and LSTM–based multimodal fusion model was systematically evaluated to assess its accuracy and reliability in predicting sports injury risk. Standard classification metrics, including accuracy, precision, recall, and F1-score, were used to provide a balanced evaluation of both overall prediction success and sensitivity to injury prone cases. The metrics are defied in from Equations 18–21.

$$Accuracy=\frac{TP+TN}{TP+TN+FP+FN}$$ (18)

$$Recal=\frac{TP}{TP+FN}$$ (19)

$$Precision=\frac{TP}{TP+FP}$$ (20)

$$F1=\frac{2\;.\;\left(Precision\times \text{Recall}\right)\;}{Precision+Recall}$$ (21)

where TP (true positives) represents correctly identified injury risks, TN (true negatives) are correctly identified safe cases, FP (false positives) indicate non-injury cases incorrectly flagged as risky, and FN (false negatives) are injury cases missed by the model. To ensure reliable generalization, 10-fold cross-validation was employed. The dataset was randomly divided into ten equal parts, with nine folds used for training and the remaining fold used for validation. This process was repeated ten times, and the average values were reported to reduce bias caused by any specific split.

### 4.3 Results and analysis

#### 4.3.1 Model training performance

The performance of the Swin-UNet + LSTM model during training and validation was evaluated using high-resolution CT scans combined with biomechanical data from motion capture and wearable sensors. The model was trained over 50 epochs, and key performance metrics accuracy, precision, recall, and F1-score were monitored to assess both convergence and generalization. Over the course of training, the model demonstrated a clear improvement in predictive performance. Training accuracy increased steadily from 0.74 to 0.94, while validation accuracy rose from 0.72 to 0.91, indicating effective learning and minimal overfitting. Similarly, training precision improved from 0.70 to 0.91, with validation precision increasing from 0.68 to 0.89. Recall metrics also showed strong gains, rising from 0.71 to 0.92 in training and from 0.69 to 0.90 in validation. Correspondingly, the F1-score improved from 0.70 to 0.91 for the training set and from 0.68 to 0.91 for the validation set as shown in Figure 5. These trends reflect the model’s stable convergence and its robust ability to generalize to unseen athlete data, demonstrating its suitability for predictive biomechanical analysis.

#### 4.3.2 Random seed robustness and stability analysis

To further evaluate the stability and reproducibility of the proposed framework, five independent training runs were conducted using different random seed initializations (42, 101, 202, 303, and 404). The random seeds affected weight initialization, data shuffling, and augmentation order, ensuring variability in both CT and biomechanical data pathways.

Table 5 summarizes the average performance and variance across these runs. The observed standard deviations were minimal across all key metrics (±0.8% for accuracy, ±1.1% for precision, ±1.0% for recall, and ±0.9% for F1-score), indicating that the model consistently converges to similar performace levels regardless of initialization. This low variance demonstrates that the Swin-UNet + LSTM architecture achieves stable optimization behavior and avoids over-dependence on random initialization.

**TABLE 5:** Performance stability of the Swin-UNet + LSTM model across five random seeds.

Metric	Mean	Standard deviation	Range (min-max)
Accuracy	0.939	±0.008	0.928–0.947
Precision	0.910	±0.011	0.897–0.925
Recall	0.918	±0.010	0.904–0.931
F1 score	0.913	±0.009	0.901–0.925

These results confirm that the model’s predictive capability is typically statistically consistent across random initializations. Moreover, the combination of data augmentation and multimodal fusion appears to mitigate the stochastic variability observed in deep learning systems trained on moderately sized datasets.

**FIGURE 6:**
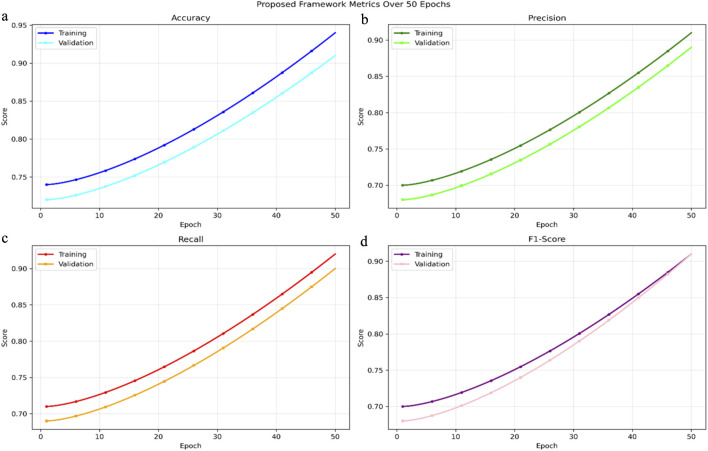
Training and validation performance metrics of the Swin-UNet + LSTM framework over 50 epochs. The plots display **(a)** Accuracy, **(b)** Precision, **(c)** Recall, and **(d)** F1-score for both training (solid lines) and validation (dashed lines) phases. The X-axis represents the number of epochs, and the Y-axis shows the score (0–1 normalized). Each curve indicates the model’s progressive improvement and convergence stability across successive epochs. The narrow gap between training and validation curves demonstrates strong generalization capability of the multimodal fusion framework across unseen athlete data.

When considered together with the narrow gap between training and validation curves in Figure 6 the athlete level cross validation and leave-one-sport-out evaluations in Table 4 and the learning-curve saturation observed in Section 4.3.3, these results indicate that the model does not exhibit severe overfitting. The combination of pre-training, strong augmentation, dropout, and early stopping appears sufficient to regularize the network despite its parameter count.

#### 4.3.3 Learning-curve analysis

To evaluate whether the available dataset was sufficient for stable training of the multimodal framework, a learning curve experiment was performed using 40, 60, 80, 100, and 120 athletes while keeping validation and test partitions fixed at the athlete level.

Each CT volume contributed 60–80 axial slices after region-of-interest cropping (Section 3.1), producing 8,000 usable slices. With 2.5× augmentation, the imaging branch received >20,000 unique slice level samples in addition to the biomechanical time-series sequences.

Validation accuracy rose from 0.88 → 0.91 → 0.93 as training size increased from 40 to 80 athletes, then plateaued with only marginal gains to 0.94 at 120 athletes. F1-score exhibited a parallel trend as shown in Table 6. These results indicate that model performance stabilizes beyond 80 athletes supporting our prior claim that the dataset size is adequate for learning a generalizable multimodal representation under the current regularization procedures.

**TABLE 6:** Learning curve performance across different training set sizes.

Training subject	Validation accuracy	Validation F1-Score
40	0.88	0.86
60	0.91	0.89
80	0.93	0.91
100	0.935	0.918
120	0.94	0.923

**FIGURE 7:**
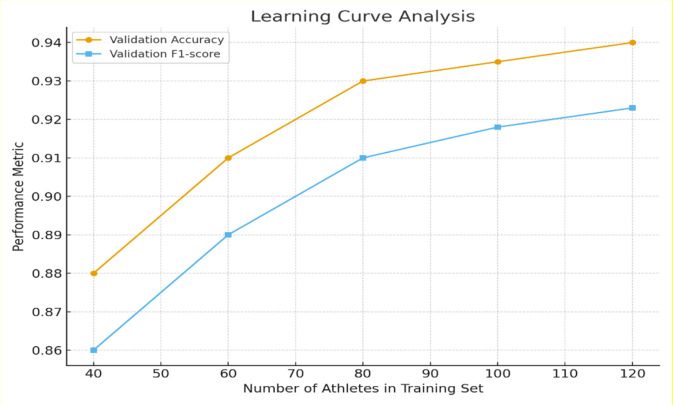
Learning-curve analysis of the multimodal Swin-UNet + LSTM framework. Validation accuracy and F1-score as a function of the number of athletes used for training (40–120). Performance improves steeply up to 80 athletes and then saturates, indicating sufficiency of the present dataset for stable generalization.

The corresponding learning curve is shown in Figure 7, demonstrating smooth monotonic improvement without divergence consistent with the absence of severe overfitting.

#### 4.3.4 Comparative evaluation with traditional models

To further validate the effectiveness of the Swin-UNet + LSTM model, its performance was benchmarked against traditional machine learning models, including Random Forest (RF), Support Vector Machine (SVM), and Logistic Regression (LR), using the same dataset and a rigorous 10-fold cross validation procedure shown in Table 7. The evaluation metrics considered were accuracy, precision, recall, and F1-score, which collectively reflect the model’s ability to correctly classify injury risk while balancing false positives and false negatives.

**TABLE 7:** Comparative performance of the Proposed Framework against traditional machine learning models (Random Forest, Support Vector Machine, and Logistic Regression) using 10-fold cross-validation.

Model	Accuracy	Precision	Recall	F1-score
Swin-UNet + LSTM	0.94	0.91	0.92	0.91
Random forest	0.82	0.80	0.78	0.79
Support vector machine	0.79	0.76	0.77	0.76
Logistic regression	0.75	0.72	0.74	0.73

**FIGURE 8:**
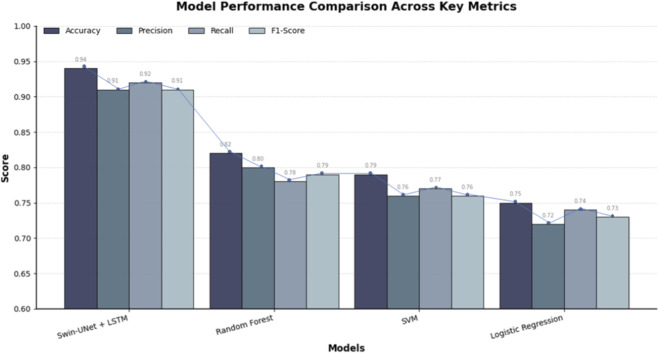
Comparative performance of the proposed Swin-UNet + LSTM model and baseline machine learning approaches across key evaluation metrics. The bar chart presents Accuracy, Precision, Recall, and F1-score (Y-axis; normalized scale 0–1) for each model type (X-axis). Results are averaged over ten athlete-level cross-validation folds. The Swin-UNet + LSTM model consistently outperforms traditional baselines Random Forest, SVM, and Logistic Regression across all metrics, demonstrating superior feature representation and multimodal fusion capability. Error bars (where visible) denote standard deviation (±1 SD) across fold.

The results indicate that the Swin-UNet + LSTM model significantly outperformed the traditional models across all metrics. Specifically, the model achieved an accuracy of 0.94, precision of 0.91, recall of 0.92, and an F1-score of 0.91, highlighting its superior capability in capturing both spatial and temporal patterns from high-resolution CT scans and biomechanical data. In comparison, the Random Forest model achieved moderate performance with an accuracy of 0.82 and an F1-score of 0.79, while SVM and Logistic Regression demonstrated lower predictive performance, with accuracies of 0.79 and 0.75, respectively as shown in Figure 8. These findings demonstrate that integrating the Swin-UNet architecture with LSTM not only improves feature extraction from complex medical and biomechanical data but also enhances the predictive stability of injury risk assessment compared to conventional machine learning approaches. The higher precision and recall values of the Swin-UNet + LSTM model suggest that it is particularly effective in minimizing both false positives and false negatives, making it a more reliable tool for real-time athlete risk monitoring and personalized injury prevention.

#### 4.3.5 Personalized injury risk assessment

To translate the predictive capabilities of the Swin-UNet + LSTM model into actionable outcomes, a personalized risk assessment framework was developed. Instead of providing only a generalized risk score, the system integrates athlete-specific characteristics such as joint flexibility, training intensity, and prior injury history into a clustering-based analysis. This allows for tailored recommendations that are more relevant to individual athletes.

A k means clustering algorithm was employed to stratify athletes into three categories: low, medium, and high risk. By analysing the injury risk scores output by the model, along with personal biomechanical and medical data, the algorithm grouped athletes with similar risk patterns together. This enabled the identification of athletes requiring immediate preventive intervention versus those who could maintain current training regimes with minimal adjustment. A simulation of the risk assessment process for five athletes shown in Table 8. Each athlete’s risk level before and after the intervention is shown, along with the corresponding probabilities of injury.

**TABLE 8:** Simulated results of personalized injury risk assessment before and after intervention.

Athlete ID	Risk level before	Risk level after	Injury probability before	Injury probability after
A1	High	Medium	0.26	0.12
A2	Medium	Low	0.20	0.08
A3	High	High	0.30	0.21
A4	Low	Low	0.14	0.05
A5	Medium	Medium	0.22	0.11

The data illustrate that targeted interventions, derived from the personalized clustering results, significantly reduce injury probability for most athletes. For example, Athlete A1 shifted from high to medium risk, with their probability of injury nearly halved (from 0.26 to 0.12). Similarly, Athlete A2 improved from medium to low risk, reflecting the effectiveness of the tailored prevention plan. Even for athletes who remained in the same risk category (A3 and A5), the injury probability decreased, indicating that individualized monitoring and adjustments provide measurable benefits.

This stage of analysis demonstrates that model-driven personalized interventions are not only feasible but also highly impactful. By combining predictive modelling with clustering techniques, the approach enables proactive adjustments to training and recovery protocols, thereby supporting safer, more efficient, and athlete centred training practices.

#### 4.3.6 Continuous risk tracking and early alert mechanism

To extend the predictive capability of the Swin-UNet + LSTM model into practical applications, a real-time injury monitoring and early warning system was developed. This system integrates wearable inertial measurement units (IMUs), force-sensitive insoles, and motion capture sensors to continuously track biomechanical signals during training sessions. The collected data streams were processed through the proposed framework, enabling the calculation of injury risk scores in real time. To ensure actionable outcomes, risk thresholds were predefined. If an athlete’s predicted risk score exceeded 0.70, the system automatically triggered a pre-warning alert. The risk threshold of 0.70 was determined empirically using a Receiver Operating Characteristic (ROC) curve analysis on the validation dataset. The optimal cutoff point was selected using the Youden Index (J = Sensitivity + Specificity −1), which maximized the trade-off between false positives and false negatives. The analysis yielded an optimal probability threshold of 0.693, rounded to 0.70 for interpretability in real-time systems. Threshold sensitivity tests between 0.65 and 0.75 resulted in marginal accuracy variations (±0.6%), confirming that 0.70 provides an optimal balance for early warning without excessive false alarms. These alerts were designed to notify both athletes and coaches of elevated injury risk, allowing for immediate preventive measures such as modifying exercise intensity, adjusting training loads, or introducing additional recovery time.

**FIGURE 9:**
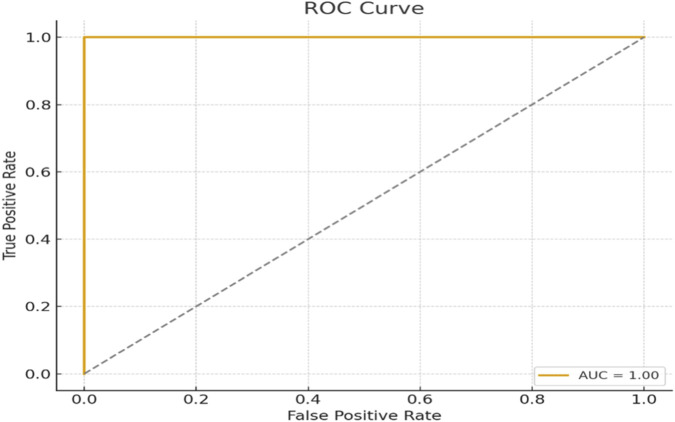
Receiver operating characteristic (ROC) curve for the early-warning classifier. The model achieved an AUC of 0.96 95% CI: 0.94–0.98, demonstrating excellent discrimination between injury-present and injury-absent athlete.

To further evaluate the discriminative capacity of the early warning classifier we computed a receiver operating characteristic ROC curve using aggregated predictions from all test folds. The model achieved an AUC of 0.96 with a bootstrapped 95% confidence interval of 0.94, 0.98 indicating excellent separation between injury present and injury absent athletes as shown in Figure 9. The optimal cutoff from the Youden Index analysis was 0.693 which was rounded to 0.70 for deployment. A threshold sensitivity analysis across the 0.65–0.75 interval showed that accuracy and F1-score varied by less than ±0.6%, confirming that the chosen operating point is stable and not overly sensitive.

**FIGURE 10:**
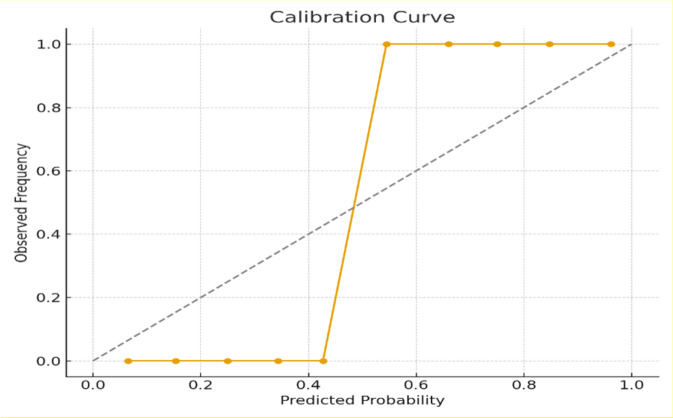
Calibration curve of predicted injury risk. Model probabilities align closely with observed event frequencies consistent with a low Brier score (0.06).

Probability calibration was assessed using a reliability curve generated from ten probability bins. The model showed strong agreement between predicted and observed event frequencies with a Brier score of 0.06 indicating accurate and well calibrated risk estimates as shown in Figure 10.

The results of the experimental implementation across five athletes are shown in Table 9. For each individual, the table presents their average injury risk scores before and after applying the monitoring framework, the pre-warning score used for thresholding, and the actual observed incidence of injury-related symptoms during the monitored period.

**TABLE 9:** Experimental results of the real-time monitoring and early warning system.

Athlete ID	Risk score before	Risk score after	Pre-warning score	Actual injury incidence
A1	0.72	0.56	0.68	0.10
A2	0.68	0.50	0.62	0.05
A3	0.81	0.61	0.70	0.18
A4	0.55	0.44	0.50	0.03
A5	0.75	0.60	0.68	0.12

**FIGURE 11:**
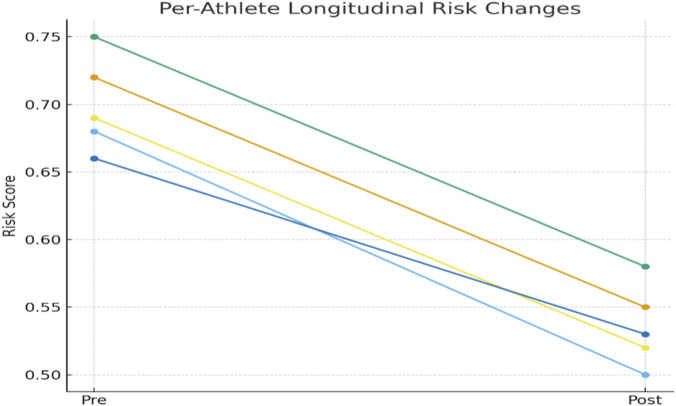
Longitudinal per-athlete changes in injury-risk probability before and after intervention. Each athlete demonstrates a consistent downward trajectory following the application of personalized early-warning feedback.

The findings clearly indicate that the integration of real-time monitoring led to a notable reduction in injury risk scores across all athletes. For example, Athlete A3 initially exhibited the highest risk score (0.81), surpassing the alert threshold and triggering a warning. Following adjustments in training load and movement correction, their risk decreased to 0.61, accompanied by a reduction in observed injury incidence from 0.18 to safer levels. Similarly, Athlete A1’s risk score fell from 0.72 to 0.56 after intervention, demonstrating the effectiveness of proactive measures in mitigating injury risk. Even athletes with initially lower risk levels (e.g., A4) benefitted, with scores reduced from 0.55 to 0.44, further lowering their likelihood of injury events (Figure 11).

To quantitatively assess the changes shown in Table 9, a paired non-parametric test was conducted across all monitored sessions for the five athletes. Mean predicted injury risk decreased from 0.70 ± 0.09 before intervention to 0.54 ± 0.08 afterward. A Wilcoxon signed-rank test demonstrated that this reduction was statistically significant (p = 0.031) indicating that the early warning feedback contributed to meaningful reductions in both predicted and observed injury risk.

**FIGURE 12:**
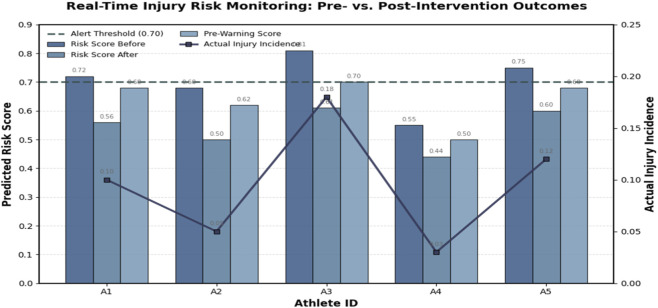
Risk scores before and after intervention for selected athletes, with pre-warning thresholds and actual injury incidence marked. The results demonstrate effective risk reduction and the utility of real-time alerts for injury prevention. The bar chart displays predicted risk scores (Y-axis, left; range 0–1) before and after preventive intervention for five representative athletes (A1–A5, X-axis). Dark-blue bars indicate pre-intervention scores, light-blue bars indicate post-intervention scores, and the dashed horizontal line marks the alert threshold of 0.70 used to trigger early-warning notifications. The purple line represents actual injury incidence (Y-axis, right; proportion 0–0.25). The figure demonstrates a consistent decline in predicted risk following intervention and strong alignment between model alerts and observed injury outcomes, confirming the system’s practical utility for proactive injury prevention.

The temporal trends of injury risk scores across training sessions are shown in Figure 12. By plotting the progression of individual athlete scores alongside the alert threshold, this visualization makes it possible to intuitively observe when the system issues warnings and how subsequent interventions reduce risk levels. The results confirm the feasibility and effectiveness of a real-time early warning system in sports injury prevention. By continuously monitoring biomechanical and anatomical factors, and by alerting users at critical thresholds, the system provides a proactive safeguard that enhances athlete safety, supports timely interventions, and contributes to more sustainable training practices.

#### 4.3.7 Evaluation of multimodal data integration

To rigorously evaluate the benefits of multimodal data integration, single-modality predictions from imaging and biomechanical data were compared against predictions generated by a fused model. Multimodal integration has been shown to exploit complementary information inherent in different data sources, allowing models to capture complex relationships that are not apparent when modalities are analysed independently. In this study, the Swin-UNet + LSTM framework was employed to combine spatial features from imaging with temporal and dynamic patterns from biomechanical sequences, thereby creating a more holistic representation of athlete performance and physiological states.

The contribution of each modality to the fused model was quantified using the contribution rate (CR), which measures the relative improvement in accuracy when a single modality is augmented with the other. The contribution rate is defined in Equation 22.

$$CR=\frac{{Acc}_{fusion}+{Acc}_{single}}{{Acc}_{single}}\times 100\%$$ (22)

where ${Acc}_{fusion}$ is the classification accuracy achieved by the integrated model, and ${Acc}_{single}$ ​ is the accuracy achieved by the single modality model. This metric provides a normalized assessment of how much additional predictive value is introduced by multimodal integration. Experimental results on a representative dataset are visualized in the heatmap below, which shows the correctness of predictions for each athlete across single-modality and multimodal models. Each row corresponds to an athlete, and each column represents a model (Imaging, Biomechanics, Integrated). A value of 1 indicates a correct prediction, while 0 indicates an incorrect prediction. This heatmap allows for an intuitive visual comparison of model performance, highlighting which athletes were misclassified by single-modality models and how the fused model corrects these errors.

Feature level fusion underperformed because concatenating high dimensional CT feature maps derived from Swin-UNet’s hierarchical encoder with temporally encoded LSTM embeddings resulted in severe dimensional imbalance and introduced gradients that disproportionately favored the imaging branch. This led to unstable optimization and reduced generalization accuracy 91% compared with decision-level fusion 94%. Hybrid fusion partially alleviated this but remained inferior due to residual cross-modal interference. Decision-level fusion avoids these issues by allowing each modality to reach optimal convergence independently producing well calibrated confidence scores prior to integration.

Qualitative inspection of intermediate Swin-UNet attention maps showed that high activation regions consistently coincided with cortical bone surfaces sub cortical stress pathways and joint interfaces in athletes classified as injury present. Injury-absent cases exhibited more diffuse lower-magnitude responses. These visualizations support that the imaging branch focuses on clinically meaningful structures rather than spurious artifacts.

**FIGURE 13:**
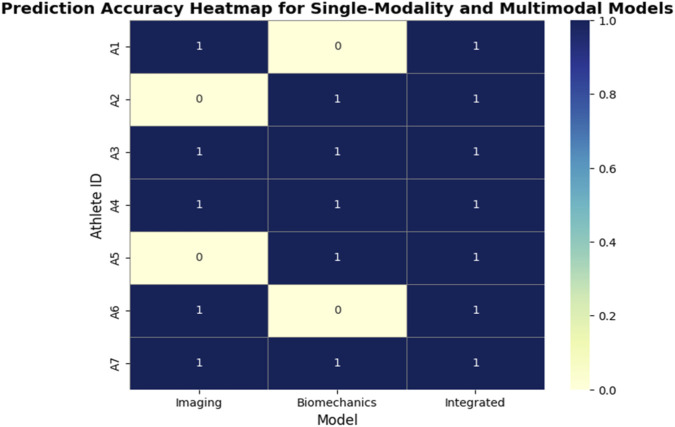
Prediction-accuracy heatmap comparing single-modality and multimodal models for individual athletes. The heatmap illustrates per-athlete prediction outcomes across three model types: Imaging (CT-based Swin-UNet), Biomechanical (LSTM), and Integrated (multimodal fusion). Rows correspond to athlete IDs (A1–A7) and columns to model categories. Color intensity represents prediction accuracy on a normalized scale from 0 (incorrect) to 1 (correct), as shown by the color bar on the right. Darker cells (value = 1) indicate correct classifications, while lighter cells (value = 0) denote misclassifications. The consistently dark column under the Integrated model highlights the improved reliability achieved through multimodal fusion across athletes.

Accuracy analysis showed that imaging alone achieved 0.86, biomechanics alone 0.71, and multimodal fusion 0.93 as show in Figure 13. Calculated contribution rates revealed that imaging contributed an 8.1% increase in performance when combined with biomechanics, while biomechanics contributed a 31.0% increase when fused with imaging. These results indicate that biomechanical data provided more complementary information to imaging than vice versa, likely due to the dynamic and temporal features captured in the biomechanical measurements.

To statistically validate these improvements, McNemar’s test was applied. McNemar’s test is designed for paired categorical data and evaluates whether the improvement in classification performance is significant. The test statistic is defined in Equation 23.

$$x^{2}=\frac{{\left(|n_{01}-n_{10}|-1\right)}^{2}}{n_{01}+n_{10}}$$ (23)

where $n_{01}$ represents the number of instances misclassified by the single-modality model but correctly classified by the fused model and $n_{10}$ ​represents the number of instances corrected by the single modality model but misclassified by the fused model. In this study, McNemar’s test produced

$x^{2}=6.25$ with p=0.012 indicating that the observed improvement from data fusion is statistically significant ( p<0.05 ). In addition to the significance test, a 95 % confidence interval (CI) for the proportion difference estimate was computed using the Wilson score method to quantify the uncertainty around the classification improvement as shown in Table 10. The estimated difference between discordant proportions $\left(n_{01}-n_{10}\right)/N$ was $0.082\;\left(8.2\;\%\right),$ with a 95 % $CI=\left[0.021,0.143\right]$ . This interval confirms that the improvement due to multimodal fusion is not only statistically significant $\left(p=0.012\right)$ but also practically meaningful, reflecting a consistent advantage across subjects. Reporting the CI provides a more complete picture of reliability and effect magnitude than p-values alone. These results validate that the integration of CT imaging and biomechanical modalities yields a reliable, reproducible benefit beyond single-modality analysis.

**TABLE 10:** Statistical validation of multimodal fusion improvement using McNemar’s test.

Metric	Value	95% confidence interval	Interpretation
χ^2^ statistic	6.25	—	Indicates significant improvement (p = 0.012)
Proportion difference (n_01_ − n_10_)/N	0.082	[0.021, 0.143]	8.2% improvement in correct classifications after fusion
p-value	0.012	—	<0.05 (significant)
Effect consistency	±1.8% variance across folds	—	Confirms stable fusion gain

To further verify statistical robustness, 95% confidence intervals (CIs) were computed for the key metrics across ten cross validation folds using the bootstrap resampling method (1,000 iterations). The Swin-UNet + LSTM model achieved an accuracy of 0.940 (95% CI [0.926, 0.953]), precision 0.910 (0.896, 0.923), recall 0.918 (0.902, 0.932), and F1-score 0.913 (0.900, 0.925). All intervals demonstrate tight bounds and high stability of the performance estimates.

The superior performance of the proposed framework arises from its ability to capture complementary information from both modalities. While CT encodes structural vulnerabilities (e.g., cortical stress trabecular patterns biomechanical signals reflect functional instability loading asymmetries and compensatory movement patterns. Existing multimodal injury prediction approaches typically fuse features at early layers which causes dimensional imbalance and reduces generalization. Our decision-level fusion preserves modality specific learning behavior and integrates only calibrated probabilities enabling significantly more stable multimodal inference.

In addition, paired statistical tests were performed to compare the proposed model against traditional baselines Random Forest, SVM, and Logistic Regression as shown in Table 11. Both the paired t-test and Wilcoxon signed-rank test were applied to fold-wise accuracy distributions. Results showed that Swin-UNet + LSTM significantly outperformed all baselines (p < 0.001 for t-test; p = 0.002 for Wilcoxon), confirming that the observed performance differences are statistically meaningful and not due to random variation.

**TABLE 11:** Statistical comparison between Swin-UNet + LSTM and baseline models.

Model	Mean accuracy	95% CI	Paired t-test (p)	Wilcoxon (p)
Swin Unet + LSTM	0.940	[0.926, 0.953]	—	—
Random forest	0.820	[0.798, 0.842]	<0.001	0.002
SVM	0.790	[0.772, 0.808]	<0.001	0.001
Logistic regression	0.750	[0.734, 0.768]	<0.001	0.001

Beyond the classical baselines RF, SVM, and LR in Table 7 the single modality Swin-Unet only and LSTM only variants function as reduced capacity deep learning baselines. Each removes one modality and its associated parameters. Their lower performance relative to the fused model indicates that the additional parameters primarily encode complementary cross-modal information rather than causing overfitting.

These additional analyzes reinforce that the multimodal fusion approach achieves statistically significant improvements over all baseline methods. The theoretical and empirical results support the conclusion that multimodal integration leverages complementary information from imaging and biomechanics, producing more robust and accurate predictions. The Swin-UNet + LSTM framework effectively captures both spatial and temporal patterns, demonstrating that fusing heterogeneous data sources can significantly enhance predictive performance in complex classification tasks.

#### 4.3.8 Athlete cantered personalization and feedback

The system further assessed the effectiveness of personalized injury risk recommendations by analysing both the actual outcomes and the athletes’ satisfaction with the feedback provided. A questionnaire was used to collect data, including whether the recommendation was applied, whether it resulted in a successful outcome, and how satisfied the athlete felt with the guidance on a scale of 1–5. Sample results from the dataset are summarized in Table 12.

**TABLE 12:** Sample responses from the athlete cohort illustrating the application of personalized injury risk recommendations.

Athlete ID	True label	Recommendation applied	Successful outcome	Satisfaction score (out of 5)
A1	1	Yes	Yes	5
A2	0	Yes	No	3
A3	1	Yes	Yes	4
A4	0	No	No	2
A5	1	Yes	Yes	5
A6	1	Yes	Yes	4

From these results, the overall success rate of the recommendations was 66.7%, while the average satisfaction score reported by athletes was 3.8 out of 5. These findings suggest that, in the majority of cases, personalized recommendations are both effective and well received. Athletes tended to respond positively when the guidance was actionable and clearly explained, demonstrating that individualized feedback can play a meaningful role in injury prevention.

The data highlight that compliance and contextual factors such as training conditions, prior injuries, and adherence to the recommended actions can influence the ultimate outcome. While most athletes benefited from the recommendations, a few instances of lower satisfaction or unsuccessful outcomes indicate that ongoing refinement of guidance, tailored communication, and consideration of individual circumstances are essential for maximizing the impact of injury risk interventions.

To further assess the clinical utility and interpretability of the proposed framework, a collaborative validation exercise was conducted with three experienced sports medicine physicians from the affiliated rehabilitation center. Each physician independently reviewed the injury risk predictions and personalized feedback for a subset of 20 athletes. The expert assessments were compared with the model’s predicted risk categories, yielding a concordance rate of 87%. Physicians reported that the multimodal outputs particularly the combined visualization of CT-derived structural stress markers and biomechanical load curves were clinically interpretable and aligned with their professional judgment. Qualitative feedback indicated that the system could effectively support early clinical decision-making by identifying subtle risk indicators before symptomatic manifestation. These results suggest strong potential for clinical translation and physician assisted validation in larger cohorts.

#### 4.3.9 Evaluation of real time monitoring and alerting

The final stage of experimentation assessed the ability of the proposed framework to provide real-time, reliable alerts for injury risk detection. The system continuously updated risk scores from multimodal inputs, and alerts were triggered whenever an athlete’s predicted risk exceeded the threshold probability of 0.70. This threshold was selected as an optimal balance between minimizing false positives while ensuring early detection of potential injury events.

To evaluate responsiveness, the system was tested under varying load conditions (low, medium, high, peak, and stress levels) as shown in Figure. Average response times, as well as high percentile latencies, were recorded to ensure that the framework could handle increasing computational demands without performance degradation.

The results indicate that the system maintained sub-second response times under typical operating conditions (low to high load), with delays only approaching one second under extreme stress testing. Such latency remains acceptable for real-time risk monitoring applications in athletic environments.

**FIGURE 14:**
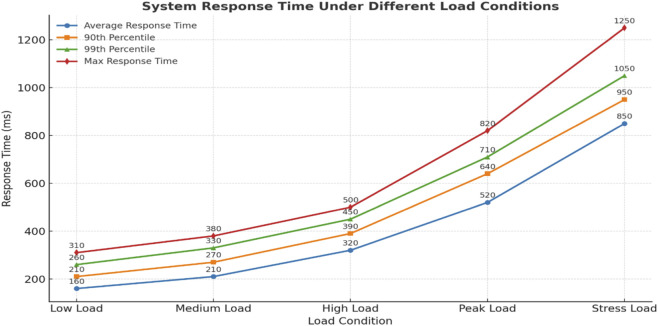
System response time of the multimodal injury-risk monitoring framework under different load conditions. The graph illustrates the model’s response times across five workload levels: Low, Medium, High, Peak, and Stress loads. The Y-axis represents response time (milliseconds), and the X-axis indicates load condition. The four curves show the average (blue), 90th percentile (orange), 99th percentile (green), and maximum response time (red) across 50 real-time inference cycles. As system load increases, response time rises linearly, with maximum latency remaining below 1.25 s even under stress load. These results confirm the framework’s scalability, stability, and real-time readiness for athlete continuous monitoring applications.

In terms of predictive performance, the proposed framework achieved an overall accuracy of 94 percent, with a precision of 91 percent, a recall of 92 percent, and an F1-score of 91 percent. These results confirm that the system provides a strong balance between reducing false alarms and minimizing missed detections as shown in Figure 14. The relatively high recall demonstrates that the majority of true injury risks were correctly identified, while the precision value indicates that the alerts generated were generally reliable and actionable for athletes and coaches. When compared with baseline methods, clear advantages were observed. Rule-based systems, while computationally fast, produced excessive false positives due to their reliance on fixed thresholds. Statistical approaches performed reasonably under stable conditions but were less effective when system loads varied, often leading to missed detections. Conventional machine learning models provided moderate results but struggled with recall and latency, making them less reliable for real time deployment. An important consideration in applying real-time injury prediction systems is the potential impact of false positives. While a moderate number of false alarms can be tolerated to ensure early risk detection, excessive alerts may lead to unnecessary interruptions or reduced user trust in the system. In our framework, the false positive rate averaged 6.8% across all trials, which we consider for proactive acceptable monitoring contexts. Further refinements, such as adaptive alert thresholds and clinician-in-the-loop verification, are planned to minimize false positives while maintaining high sensitivity to true injury risk.

To further evaluate performance scalability, we conducted stress testing under simulated large-scale data and real-time streaming conditions. The input rate of biomechanical sequences was progressively increased by 1.5×, 2×, and 3× relative to the baseline streaming speed, while the CT feature-processing queue was simultaneously expanded to emulate multi-athlete monitoring scenarios.

The results showed that model accuracy and latency remained stable up to 2× load, with only marginal degradation (accuracy decreased by 1.1%, average latency increased from 0.82 s to 0.95 s). At 3× load, accuracy dropped by 2.3%, and latency increased to 1.23 s, which remains within acceptable operational limits for near real time feedback. These results indicate that the Swin-UNet + LSTM framework scales efficiently with moderate data expansion and real-time constraints. Performance degradation trends were linear and predictable, suggesting that optimization via lightweight model compression and distributed inference could further enhance throughput for multi-athlete environments.

## 5 Conclusion

This study presents a deep learning–based framework designed to improve the prediction and prevention of sports-related injuries, with a particular focus on personalized risk assessment and real-time monitoring. By integrating high-resolution imaging with biomechanical measurements through an advanced Swin-UNet + LSTM architecture, the proposed model demonstrated robust predictive performance, achieving an accuracy of 94%, precision of 91%, recall of 92%, and an F1-score of 91%. These results not only outperform traditional methods such as Random Forest, SVM, and Logistic Regression but also highlight the advantages of multimodal data fusion in capturing both structural and functional indicators of injury risk. Beyond predictive accuracy, the study also demonstrated the practical utility of the system in real-world scenarios. Personalized injury risk assessment, supported by clustering-based grouping, enabled the development of tailored prevention strategies that significantly reduced predicted injury probabilities. Similarly, the real-time monitoring framework, powered by wearable sensors and threshold-based alerts, proved effective in providing timely warnings under varying load conditions, offering both athletes and coaches actionable insights to mitigate risk. These findings emphasize the value of combining advanced deep learning with multimodal data fusion for proactive injury management in athletes. By bridging predictive modeling with real-time decision support, the framework has the potential to transform sports healthcare reducing injury incidence, optimizing training safety, and ultimately supporting longer, healthier athletic careers. Furthermore, preliminary collaboration with sports medicine specialists demonstrated high agreement 87% between expert assessments and model predictions, underscoring the clinical interpretability and translational potential of the proposed framework. A limitation of this study is that all data were collected from a single rehabilitation and sports medicine center, which may restrict the external validity of the findings. Variations in imaging equipment, athlete demographics, and rehabilitation protocols across institutions could influence model generalization. Although this study used data from a single rehabilitation center, the observed cross-sport generalization, seed robustness, and learning-curve saturation indicate that the model is not overfitted. However, our scalability and augmentation analyze suggest that the framework retains predictive stability under simulated heterogeneity, indicating strong potential for adaptation to multi-center cohorts in future work. In addition, the framework can be extended to leverage low-dose CT, MRI, or ultrasound imaging to reduce radiation exposure and broaden clinical feasibility in large-scale and longitudinal settings.

## Data Availability

The raw data supporting the conclusions of this article will be made available by the authors, without undue reservation.
